# Dietary Exposure to Pesticide Residues from Commodities Alleged to Contain the Highest Contamination Levels

**DOI:** 10.1155/2011/589674

**Published:** 2011-05-15

**Authors:** Carl K. Winter, Josh M. Katz

**Affiliations:** Department of Food Science and Technology, University of California, One Shields Avenue, Davis, CA 95616, USA

## Abstract

Probabilistic techniques were used to characterize dietary exposure of consumers to pesticides found in twelve commodities implicated as having the greatest potential for pesticide residue contamination by a United States-based environmental advocacy group. Estimates of exposures were derived for the ten most frequently detected pesticide residues on each of the twelve commodities based upon residue findings from the United States Department of Agriculture's Pesticide Data Program. All pesticide exposure estimates were well below established chronic reference doses (RfDs). Only one of the 120 exposure estimates exceeded 1% of the RfD (methamidophos on bell peppers at 2% of the RfD), and only seven exposure estimates (5.8 percent) exceeded 0.1% of the RfD. Three quarters of the pesticide/commodity combinations demonstrated exposure estimates below 0.01% of the RfD (corresponding to exposures one million times below chronic No Observable Adverse Effect Levels from animal toxicology studies), and 40.8% had exposure estimates below 0.001% of the RfD. It is concluded that (1) exposures to the most commonly detected pesticides on the twelve commodities pose negligible risks to consumers, (2) substitution of organic forms of the twelve commodities for conventional forms does not result in any appreciable reduction of consumer risks, and (3) the methodology used by the environmental advocacy group to rank commodities with respect to pesticide risks lacks scientific credibility.

## 1. Introduction

Since 1995, the Environmental Working Group (EWG), a United States-based environmental advocacy organization, has developed an annual list of fruits and vegetables, frequently referred to as the “Dirty Dozen,” suspected of having the greatest potential for contamination with residues of pesticides. The EWG cautions consumers to avoid conventional forms of these fruits and vegetables and recommends that consumers purchase organic forms of these commodities to reduce their exposure to pesticide residues. The annual release of the report has traditionally generated newspaper, magazine, radio, and television coverage, and the report is considered to be quite influential in the produce purchasing decisions of millions of Americans.

In June 2010, the EWG released its most recent “Dirty Dozen” list [1]. Topping the list as the most contaminated commodity was celery, followed by peaches, strawberries, apples, blueberries, nectarines, bell peppers, spinach, cherries, kale, potatoes, and grapes (imported). According to an EWG news release, “*consumers can lower their pesticide consumption by nearly four-fifths by avoiding conventionally grown varieties of the 12 most contaminated fruits and vegetables*” [2].

It is unclear how the EWG could make such a statement since the methodology used to rank the various fruits and vegetables did not specifically quantify consumer exposure to pesticide residues in such foods. Instead, the methodology provided six separate indicators of contamination, including (1) percentage of samples tested with detectable residues, (2) percentage of samples with two or more pesticides detected, (3) average number of pesticides found on a single sample, (4) average amount of all pesticides found, (5) maximum number of pesticides found on a single sample, and (6) total number of pesticides found on the commodity [1]. Each of these indicators was normalized among the 49 most frequently consumed fruits and vegetables, and a total score was developed to form the basis for the rankings. Since none of these indicators specifically considered exposure (the product of food consumption and residue levels), it is difficult to see how the EWG could substantiate the claim that consumers could lower their pesticide consumption by nearly four-fifths by avoiding conventional forms of the “Dirty Dozen” commodities. Additionally, the toxicological significance of consumer exposure to pesticides in the diet is also not addressed through an appropriate comparison of exposure estimates with toxicological endpoints such as the reference dose (RfD) or the acceptable daily intake (ADI).

To more accurately assess the potential health impacts from consumer exposure to pesticide residues from the “Dirty Dozen” commodities, this study utilized a probabilistic modeling approach to estimate exposures. The exposure estimates were then compared with toxicological endpoints to determine the health significance of such exposures.

## 2. Materials and Methods

The EWG rankings were derived from the results of residue findings of the United States Department of Agriculture (USDA) Pesticide Data Program (PDP) and the United States Food and Drug Administration (FDA) Pesticide Program Residue Monitoring from 2000 to 2008 [1, 3, 4]. The PDP is more appropriate for risk assessment as it is not developed for enforcement, provides residue findings for produce in ready-to-eat forms (i.e., washed or peeled), includes many more samples than the FDA program, and relies upon more sensitive analytical methods. As a result, our study relied entirely upon results from the most recent PDP data collected from 2004 to 2008.

To estimate exposures to pesticides from the “Dirty Dozen” commodities, PDP data was accessed for each commodity using the most recent year of data collection.  Table 1 provides a summary of the most recent sample collections for each of the twelve commodities by the PDP and the number of samples taken.

PDP data were analyzed to identify the ten most frequently detected pesticides on each of the twelve commodities. A total of 120 separate residue files were generated, corresponding to specific files for each of the ten pesticides on each of the twelve commodities. Each residue file consisted of sample-specific findings (both detections and nondetections) for all residue determinations. Residue findings considered as nondetections were assigned a value of zero, using the same approach taken by Katz and Winter [5], rather than using the much more conservative approach of considering nondetectable residues as being to one-half of the detection limits. Exposure estimates were made using LifeLine probabilistic modeling software (LifeLine software version 5.0, Annandale, VA, http://www.thelifelinegroup.org/). This software is publicly available and uses probabilistic techniques to model exposure and risks for the general population or selected populations to chemicals in food, water, and in the home environment. The model generates populations of simulated individuals, and daily exposures are calculated for each individual on the basis of food consumption (derived from the 1994–96 and 1998 USDA's Continuing Survey of Food Intakes by Individuals) and pesticide residue levels.

Exposure estimates made in this study used an approach similar to that used by Katz and Winter [5] to differentiate exposures to pesticide residues in imported and domestic fruits and vegetables. In this present study, individual runs of 2000 composite individuals were made for each of the 120 residue files. Estimates of lifetime mean daily exposure for each of the pesticides on each of the commodities were developed.

To determine the toxicological significance of such exposures, estimates were compared with chronic RfDs developed by the United States Environmental Protection Agency (EPA). The chronic RfD represents an estimate of the amount of a chemical a person could be exposed to on a daily basis throughout the person's lifetime that is likely to be without an appreciable risk of harm [6]. For a handful of pesticides identified for which RfDs had not been developed, ADI values, which are analogous to RfDs, were used as substitutes and are denoted in Tables 2, 3, 4, 5, 6, 7, 8, 9, 10, 11, 12, and 13. Most of the ADI values were also derived from lists compiled by the EPA.

## 3. Results and Discussion

The mean exposures for the top ten pesticides detected on each of the twelve commodities are provided and compared with the RfDs in Tables 2–13.

Results demonstrate that the RfD values for each of the pesticides exceed the mean exposure estimates in all cases and that the RfDs were more than 1000 times higher than the exposure estimates in more than 90 percent of the comparisons. Such findings suggest that the potential consumer risks from exposure to the most frequently detected pesticides on the “Dirty Dozen” list of foods are negligible and cast doubts as to how consumers avoiding conventional forms of such produce items are improving their health status.

The highest relative exposure for a pesticide/commodity combination was for the organophosphate insecticide methamidophos on bell peppers. The RfD for methamidophos was still 49.5 times higher than the exposure estimate, indicating a large measure of consumer protection. It should be pointed out that the chronic RfD for methamidophos (0.05 *μ*g/kg/day) [7] is far lower than any other pesticide RfD considered in this study, and this low value seems anomalous given the lower cholinesterase-inhibiting potential of methamidophos relative to other organophosphate insecticides. Ethyl parathion, for example, is considered to be far more toxic and a much more potent inhibitor of cholinesterase than methamidophos. The EPA has not established an RfD for ethyl parathion, but the World Health Organization has established an ADI for ethyl parathion of 5 *μ*g/kg/day, or 100 times higher than the RfD for methamidophos. Regardless of the unusually low RfD for methamidophos, an exposure of 49.5 times lower than the RfD still represents an exposure 49,500 times lower than exposures to methamidophos in laboratory animals that still have not resulted in any adverse health effects. The RfD for methamidophos uses a 1,000-fold uncertainty factor when extrapolating from the results of the most sensitive animal study (a one-year dog feeding study) to determine acceptable levels for human exposure [7].

For three commodities—blueberries, cherries, and kale—the RfD was more than 30,000 times higher than the exposure estimates for all of the ten most frequently detected pesticides on those commodities. Given these findings, the inclusion of blueberries, cherries, and kale on the “Dirty Dozen” list is not justified.

Findings relating exposure estimates for all pesticide/commodity combinations to RfDs are summarized in Table 14. Only one of the 120 exposure estimates exceeded 1% of the RfD (methamidophos on bell peppers at 2% of the RfD), and only seven exposure estimates (5.8 percent) exceeded 0.1% of the RfD. Three quarters of the pesticide/commodity combinations demonstrated exposure estimates below 0.01% of the RfD, and 40.8% had exposure estimates below 0.001% of the RfD. To put this in perspective, exposure at 0.01% (one ten-thousandth) of the RfD represents an exposure one *million* times lower than the No Observable Adverse Effect Level (the highest amount given to the most sensitive animal species on a daily basis), assuming that the typical 100-fold uncertainty factor is used [6]. Such exposures are *de minimus* in terms of potential human health effects.

The methodology used to create the “Dirty Dozen” list does not appear to follow any established scientific procedures. Only one of the six indicators used by the EWG crudely considers the amount of pesticide residue detected on the various commodities, and that indicator fails to relate exposures to such residues with established health criteria. Another indicator considers the percentage of samples found to be positive for pesticide residues. The remaining four indicators seem related as all appear to focus upon the existence of residues of multiple pesticides (percent of samples with two or more pesticides, average number of pesticides found on a single sample, maximum number of pesticides found on a single sample, and total number of pesticides found on the commodity) which suggests that the commodity rankings are significantly skewed to reflect instances of multiple residues. While research has demonstrated that the toxicity of a single chemical may be modulated by the presence of another chemical, such effects still require exposure to the modulating chemical to be at a level high enough (above a threshold dose) to cause a biological effect. Results from this study strongly suggest that consumer exposures to the ten most common pesticides found on the “Dirty Dozen” commodities are several orders of magnitude below levels required to cause any biological effect. As a result, the potential for synergistic effects resulting from pesticide combinations is negligible, and the EWG methodology which skews rankings due to the presence of multiple residues is not justified. The EWG methodology also does not appear to be capable of justifying the claim that “*consumers can lower their pesticide consumption by nearly four-fifths by avoiding conventionally grown varieties of the 12 most contaminated fruits and vegetables*” since no effort to quantify consumer exposure was made.

It should also be mentioned that consumption of organic produce should not be equated with consumption of pesticide-free produce. Winter and Davis [8] summarized pesticide monitoring results from the PDP, the California Department of Pesticide Regulation, the Consumers Union, and a study in Belgium. While conventional produce was between 2.9 and 4.8 times more likely to contain detectable pesticide residues than organic produce, samples of organic produce frequently contained residues. The PDP data, in fact, indicated that 23 percent of organic food samples tested positive for pesticide residues.

In summary, findings conclusively demonstrate that consumer exposures to the ten most frequently detected pesticides on EWG's “Dirty Dozen” commodity list are at negligible levels and that the EWG methodology is insufficient to allow any meaningful rankings among commodities. We concur with EWG President Kenneth Cook who maintains that “*We recommend that people eat healthy by eating more fruits and vegetables, whether conventional or organic*” [1], but our findings do not indicate that substituting organic forms of the “Dirty Dozen” commodities for conventional forms will lead to any measurable consumer health benefit.

## Figures and Tables

**Table 1:** Number of samples analyzed by PDP for pesticide residues for each of the twelve commodities studied and the most recent year of sample collection.

	2004	2005	2007	2008
Celery	—	—	—	741
Blueberries	—	—	—	726
Kale	—	—	—	318
Nectarines	—	—	—	672
Peaches	—	—	—	616
Potatoes	—	—	—	744
Spinach	—	—	—	747
Strawberries	—	—	—	741
Cherries	—	—	419	—
Apples	—	743	—	—
Grapes (imported)	—	367	—	—
Bell peppers	558	—	—	—

**Table 2:** Exposure estimates of the ten most frequently detected pesticides on apples.

Pesticide	Mean exposure (*μ*g/kg/day)	Reference dose (*μ*g/kg/day)	Ratio—reference dose to mean exposure
Acetamiprid	0.00389	100	25700
Azinphos-Methyl	0.00488	5*	1020
Carbaryl	0.000795	100	126000
Carbendazim	0.00127	10	7870
Diphenylamine	0.12	25	208
Fenpropathrin	0.0017	25	14700
Imidacloprid	0.000202	57	282000
*ο*-Phenylphenol	0.000637	20	31400
Phosmet	0.003	20	6670
Thiabendazole	0.127	100*	787

*Acceptable daily intake used.

**Table 3:** Exposure estimates of the ten most frequently detected pesticides on bell peppers.

Pesticide	Mean exposure (*μ*g/kg/day)	Reference dose (*μ*g/kg/day)	Ratio—reference dose to mean exposure
Acephate	0.00269	4	1490
Carbendazim	0.000225	10*	44400
Chlorpyrifos	0.00185	3	1620
Dicofol	0.00042	2*	4760
Endosulfan	0.00021	6	28600
Imidacloprid	0.000442	57	129000
Metalaxyl	0.000334	74	222000
Methamidophos	0.00101	0.05	49.5
Oxamyl	0.000223	25	112000
Thiabendazole	0.00000547	100	18300000

*Acceptable daily intake used.

**Table 4:** Exposure estimates of the ten most frequently detected pesticides on blueberries.

Pesticide	Mean exposure (*μ*g/kg/day)	Reference dose (*μ*g/kg/day)	Ratio—reference dose to mean exposure
Azoxystrobin	0.0000646	180	2790000
Boscalid	0.00118	218	185000
Carbaryl	0.00011	100	909000
Carbendazim	0.000143	10*	69900
Fenbuconazole	0.0000126	300	23800000
Fludioxonil	0.000103	30	291000
Imidacloprid	0.0000178	57	3200000
Iprodione	0.000413	40	96900
Phosmet	0.000244	20	82000
Pyraclostrobin	0.00027	30	111000

*Acceptable daily intake used.

**Table 5:** Exposure estimates of the ten most frequently detected pesticides on celery.

Pesticide	Mean exposure (*μ*g/kg/day)	Reference dose (*μ*g/kg/day)	Ratio—reference dose to mean exposure
Acephate	0.00131	4	3050
Acetamiprid	0.0000997	100	1000000
Azoxystrobin	0.000675	180	267000
Cyromazine	0.000313	7.5	24000
Dicloran	0.00507	30*	5920
Imidacloprid	0.0000843	57	676000
Linuron	0.000724	2	2760
Malathion	0.000809	20	24700
Methamidophos	0.0000788	0.05	635
Permethrin	0.000693	50	72200

*Acceptable daily intake used.

**Table 6:** Exposure estimates of the ten most frequently detected pesticides on cherries.

Pesticide	Mean exposure (*μ*g/kg/day)	Reference dose (*μ*g/kg/day)	Ratio—reference dose to mean exposure
Azinphos-Methyl	0.0000485	5*	103000
Bifenthrin	0.0000169	15	888000
Boscalid	0.000357	218	611000
Carbaryl	0.000219	100	457000
Imidacloprid	0.0000956	57	596000
Myclobutanil	0.000131	30*	229000
Pyraclostrobin	0.000127	30	236000
Quinoxyfen	0.0000522	200*	3830000
Tebuconazole	0.000937	30	32000
Trifloxystrobin	0.0000915	100*	1090000

*Acceptable daily intake used.

**Table 7:** Exposure estimates of the ten most frequently detected pesticides on grapes (imported).

Pesticide	Mean exposure (*μ*g/kg/day)	Reference dose (*μ*g/kg/day)	Ratio—reference dose to mean exposure
Captan	0.00314	130	41400
Carbaryl	0.000887	100	113000
Chlorpyrifos	0.00073	3	4110
Cyprodinil	0.00612	37.5	6130
Fludioxonil	0.00279	30	10800
Folpet	0.000161	100	621000
Imidacloprid	0.00124	57	46000
Iprodione	0.00612	40	6540
Myclobutanil	0.00061	30*	49200
Tebuconazole	0.000409	30	73300

*Acceptable daily intake used.

**Table 8:** Exposure estimates of the ten most frequently detected pesticides on kale.

Pesticide	Mean exposure (*μ*g/kg/day)	Reference dose (*μ*g/kg/day)	Ratio—reference dose to mean exposure
Acetamiprid	0.00097	100	103000
Azoxystrobin	0.000231	180	779000
Boscalid	0.0000823	218	2650000
Cypermethrin	0.000278	10	36000
DCPA	0.000106	10	94300
DDE	0.0000122	0.5	41000
Imidacloprid	0.00012	57	475000
Indoxacarb	0.0000965	20	207000
Methoxyfenozide	0.000372	200	538000
Pyraclostrobin	0.000134	30	224000

**Table 9:** Exposure estimates of the ten most frequently detected pesticides on nectarines.

Pesticide	Mean exposure (*μ*g/kg/day)	Reference dose (*μ*g/kg/day)	Ratio—reference dose to mean exposure
Azinphos-Methyl	0.000196	5*	25500
Carbaryl	0.0000627	100	1590000
Chlorpyrifos	0.0000268	3	112000
Fenhexamid	0.00105	200*	190000
Fludioxonil	0.00403	30	7440
Formetanate hydrochloride	0.000174	2*	11500
Iprodione	0.00966	40	4140
Phosmet	0.00019	20	105000
Propiconazole	0.000179	13	72600
Trifloxystrobin	0.0000024	100*	41700000

*Acceptable daily intake used.

**Table 10:** Exposure estimates of the ten most frequently detected pesticides on peaches.

Pesticide	Mean exposure (*μ*g/kg/day)	Reference dose (*μ*g/kg/day)	Ratio—reference dose to mean exposure
Azinphos-Methyl	0.00194	5*	2580
Boscalid	0.000866	218	252000
Chlorpyrifos	0.000228	3	13200
Cyhalothrin	0.000227	5	22000
Fludioxonil	0.0228	30	1320
Formetanate hydrochloride	0.00351	2*	570
Iprodione	0.047	40	851
Methoxyfenozide	0.000531	100	188000
*ο*-Phenylphenol	0.000285	20*	70200
Phosmet	0.00288	20	6940

*Acceptable daily intake used.

**Table 11:** Exposure estimates of the ten most frequently detected pesticides on potatoes.

Pesticide	Mean exposure (*μ*g/kg/day)	Reference dose (*μ*g/kg/day)	Ratio—reference dose to mean exposure
Aldicarb sulfate	0.000327	1**	3060
Azoxystrobin	0.00036	180	500000
Boscalid	0.000104	218	2100000
Chlorpropham	0.322	200	621
Clothianidin	0.000064	10	156000
Flutolanil	0.000148	60	405000
Imidacloprid	0.000467	57	122000
*ο*-Phenylphenol	0.000404	20*	49500
Thiabendazole	0.00343	100*	29200
Thiamethoxam	0.0000626	13	208000

*Acceptable daily intake used.

**Aldicarb metabolite; used reference dose for aldicarb.

**Table 12:** Exposure estimates of the ten most frequently detected pesticides on spinach.

Pesticide	Mean exposure (*μ*g/kg/day)	Reference dose (*μ*g/kg/day)	Ratio—reference dose to mean exposure
Boscalid	0.0000428	218	5090000
Cyfluthrin	0.000965	25	25900
Cypermethrin	0.00632	10	1580
DDE	0.00014	0.5	3570
Imidacloprid	0.00102	57	55900
Methoxyfenozide	0.000927	100	108000
Omethoate	0.000181	0.2	1100
Permethrin	0.0144	50	3470
Pyraclostrobin	0.000331	30	90600
Spinosad	0.000685	30*	43800

*Acceptable daily intake used.

**Table 13:** Exposure estimates of the ten most frequently detected pesticides on strawberries.

Pesticide	Mean exposure (*μ*g/kg/day)	Reference dose (*μ*g/kg/day)	Ratio—reference dose to mean exposure
Bifenthrin	0.000945	15	15900
Boscalid	0.00351	218	62100
Captan	0.0159	130	8180
Cyprodinil	0.00278	37.5	13500
Fenhexamid	0.00275	57	20700
Fludioxonil	0.0012	30	25000
Malathion	0.000418	20	47800
Myclobutanil	0.000723	30*	41500
Pyraclostrobin	0.00161	30	18600
Pyrimethanil	0.00623	200*	32100

*Acceptable daily intake used.

**Table 14:** Exposure estimates relative to reference doses (RfD) for ten most frequently detected pesticides on twelve foods.

Food	EWG rank	>RfD	10% to 100% of RfD	1% to 10% of RfD	0.1% to 1% of RfD	0.01% to 0.1% of RfD	0.001% to 0.01% of RfD	0.0001% to 0.001% of RfD	<0.0001% of RfD
Celery	1	0	0	0	1	3	3	2	1
Peaches	2	0	0	0	2	3	3	2	0
Strawberries	3	0	0	0	0	1	9	0	0
Apples	4	0	0	0	2	3	3	2	0
Blueberries	5	0	0	0	0	0	3	4	3
Nectarines	6	0	0	0	0	2	3	3	2
Bell peppers	7	0	0	1	0	3	2	3	1
Spinach	8	0	0	0	0	4	4	1	1
Cherries	9	0	0	0	0	0	1	7	2
Kale	10	0	0	0	0	0	3	6	1
Potatoes	11	0	0	0	1	1	2	5	1
Grapes (imported)	12	0	0	0	0	3	5	2	0

Total		0	0	1	6	23	41	37	12
